# The Role of Immediate Ultrasound-Guided Fine-Needle Aspiration Cytology by Specialist Head and Neck Surgeons in a One-Stop Clinic: A Single-Centre Retrospective Analysis

**DOI:** 10.7759/cureus.97968

**Published:** 2025-11-27

**Authors:** Hisham Higazy, Renee Defreitas, India Jacklin-Chatha, Soo Oh, Syed Farhan Ahsan

**Affiliations:** 1 Otolaryngology, New Cross Hospital, The Royal Wolverhampton National Health Service (NHS) Trust, Wolverhampton, GBR; 2 Otolaryngology - Head and Neck Surgery, New Cross Hospital, The Royal Wolverhampton National Health Service (NHS) Trust, Wolverhampton, GBR

**Keywords:** head and neck cancer (h&n cancer), head and neck ultrasound, one-stop clinic, otolaryngology head and neck, treatment plan, ultrasound-guided fine-needle aspiration cytology

## Abstract

Background

Ultrasound-guided fine-needle aspiration cytology (US-FNAC) is a minimally invasive diagnostic technique widely used for evaluating head and neck masses. In most centres, these procedures are performed by radiologists; however, increasing service demands have resulted in delays and diagnostic backlogs, with potential for adverse effects on outcomes. A solution to this could be the use of US-FNAC performed by specialist head and neck surgeons in a one-stop neck lump clinic. Limited data exists in the current literature on US-FNAC led by head and neck surgeons.

Objective

To assess the adequacy and effectiveness of US-FNAC samples collected by head and neck surgeons in a dedicated neck lump clinic. Primary outcomes were diagnostic yield, turnaround time, and the need for subsequent imaging.

Methods

A retrospective review was conducted over a 38-month period at a single National Health Service (NHS) centre. A total of 129 patients presenting with neck lumps, excluding thyroid cases, were included. Data collected included US-FNAC sample adequacy, cytology result turnaround time, and the rate of subsequent imaging following the procedure.

Results

Of the 129 patients, 85 (66%) yielded adequate samples for cytological diagnosis. Among these, 58 (68%) were diagnostic, while 27 (32%) were non-diagnostic and required further imaging. The mean turnaround time from US-FNAC to cytology report was five calendar days, equivalent to three working days. Subsequent imaging was performed in 116 cases (89%), primarily for patients with inadequate or non-diagnostic results.

Conclusion

Immediate US-FNAC performed by specialist head and neck surgeons within a one-stop clinic achieves high diagnostic adequacy with minimal delay. This approach supports timely multidisciplinary decision-making, optimises the diagnostic pathway, and offers a feasible solution to the increasing demand on radiology departments for head and neck cancer diagnostics.

## Introduction

Ultrasound-guided fine needle aspiration cytology (US-FNAC) is a well-established diagnostic modality for evaluating head and neck masses. Its minimally invasive nature and high diagnostic accuracy make it an essential tool for distinguishing benign from malignant lesions, thereby enabling early diagnosis and guiding subsequent management.

Traditionally, US-FNAC is performed by radiologists. However, the potential role of head and neck surgeons in performing these procedures has received limited attention, particularly within structured one-stop neck lump clinic settings.

The one-stop neck lump clinic model, in which patient consultation, ultrasound evaluation, US-FNAC, and initial cytopathological assessment occur during a single visit, offers an efficient and patient-centred diagnostic pathway.

This study aims to evaluate the performance of immediate US-FNAC conducted by specialist head and neck surgeons within a one-stop clinic. The primary objectives are to assess sample adequacy, diagnostic yield, and evaluating turnaround time. The secondary objectives include determining the proportion of patients requiring further imaging.

## Materials and methods

Study design and setting

This retrospective observational study was conducted at the Shrewsbury and Telford Hospital National Health Service (NHS) Trust over a 38-month period (November 2014 to February 2018). The dedicated neck lump clinic operated under a one-stop model, allowing patients to undergo clinical consultation, ultrasound examination, and US-FNAC during a single visit.

All US-FNAC procedures were performed by a specialist head and neck surgeon (Mr Syed Farhan Ahsan), who has extensive experience in the assessment and management of neck lumps.

Patient selection

A total of 129 patients presenting with neck lumps were included in the study. Lesions were characterised according to size, location, and ultrasonographic appearance (solid, cystic, or mixed). Thyroid lesions were excluded to maintain diagnostic focus. Patients were selected for US-FNAC based on clinical suspicion (symptoms and clinical examination).

Patients with incomplete records, missing cytology reports, or those who did not undergo US-FNAC during the same clinic visit were excluded.

Data collected included sample adequacy, diagnostic yield, cytology report turnaround time and the frequency and indications for post-FNAC imaging.

Ultrasound-guided FNAC procedure

US-FNAC was performed under real-time ultrasound guidance using a 23- or 25-gauge needle. Multiple passes (typically two to three) were made to maximise cellular yield. Samples were immediately fixed and transported for cytopathological evaluation.

Sample adequacy was defined in accordance with the national cytopathology standards: the presence of sufficient cellular material to permit diagnostic categorisation. Cytology was reported using the Royal College of Pathologists (RCPath) Head and Neck Cytology classification system. All samples were reviewed by a single experienced consultant cytopathologist to maintain consistency.

Data handling

Missing data were handled using a complete-case approach; no imputation was performed. Cases with unavailable cytology results or missing imaging data were excluded from the final analysis.

Data analysis

The following variables were assessed:

US-FNAC adequacy: Whether the sample contained sufficient material for definitive cytological interpretation.

Diagnostic yield: Proportion of FNACs providing a specific diagnosis.

Turnaround time: Measured from the date of US-FNAC to the date the cytology report was issued.

Subsequent imaging: The proportion of patients requiring additional imaging (ultrasound, CT, or MRI) following FNAC due to non-diagnostic results, suspicious features, or for further planning of management.

Descriptive statistics were generated using Microsoft Excel (Version 16.77; Microsoft, Redmond, WA). Continuous variables, such as turnaround time, were expressed as mean±standard deviation (SD), while categorical variables, including FNAC adequacy, diagnostic yield, and subsequent imaging, were reported as frequencies and percentages (% and n). Statistical significance was defined as p<0.05. No formal comparative statistical testing was performed due to the retrospective and observational nature of the study.

## Results

Ultrasound-guided FNAC adequacy

**Figure 1:**
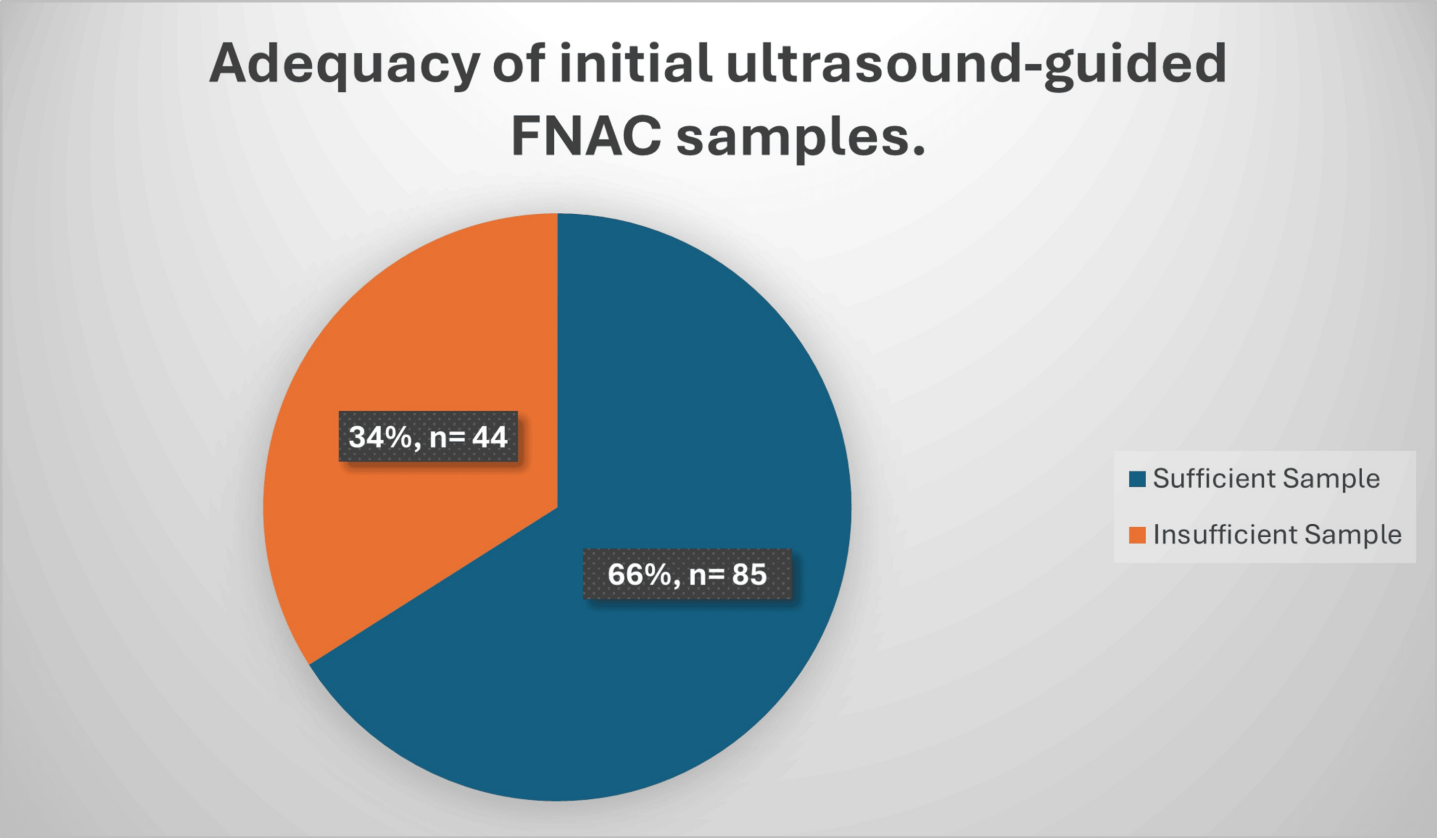
Adequacy of initial ultrasound-guided FNAC samples. Data presented as percentage (%) and number (n); statistical significance considered at p<0.05.

A total of 129 patients underwent US-FNAC. Adequate cytological samples were obtained in 66.0% (n=85), while 34.0% (n=44) yielded insufficient initial samples and required repeat US-FNAC performed by a radiographer (Figure 1).

**Figure 2:**
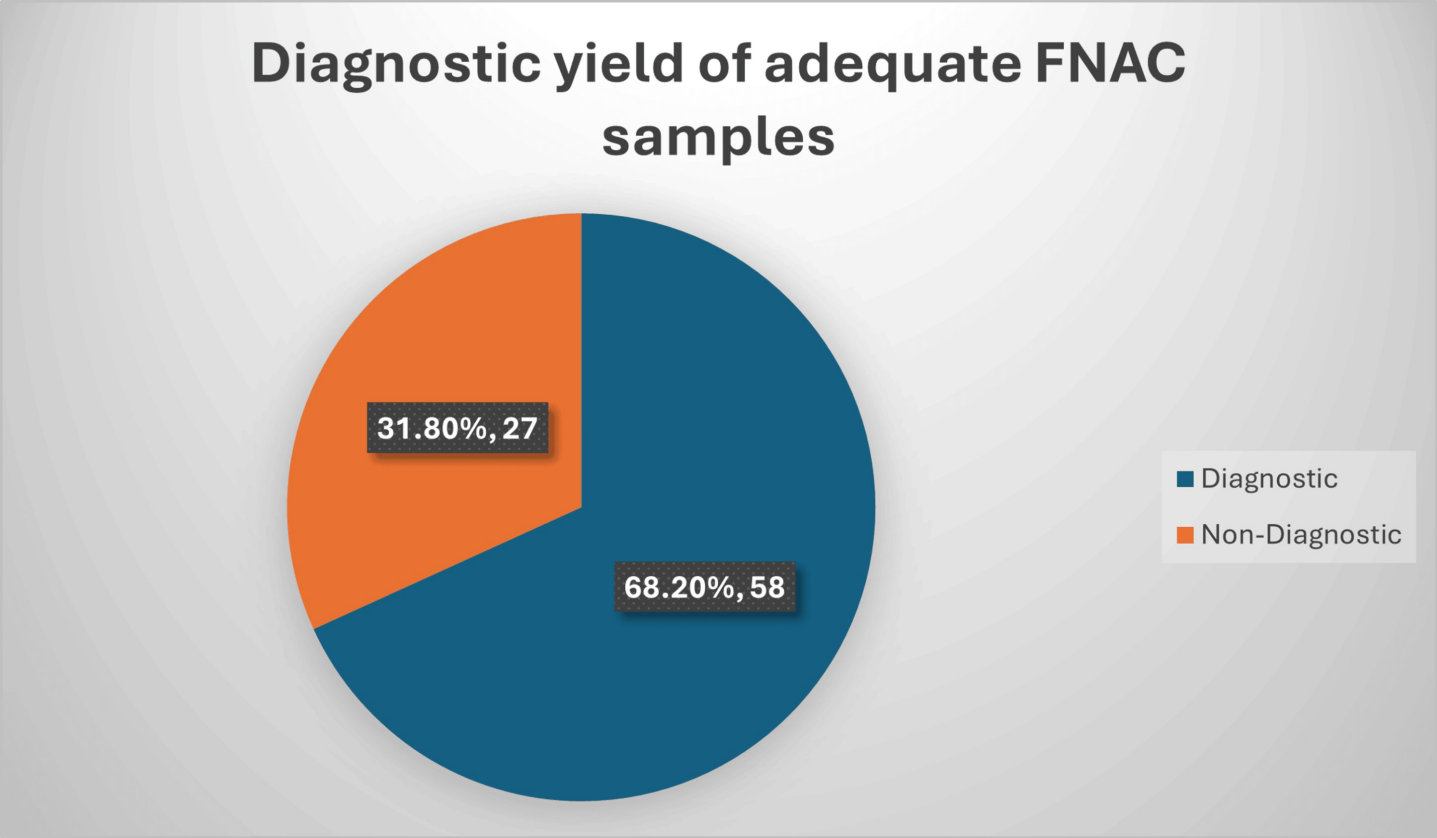
Diagnostic yield of adequate FNAC samples Data presented as percentage (%) and number (n); statistical significance considered at p<0.05.

Among patients with adequate initial FNAC samples (n=85), 68.2% (n=58) were diagnostic, providing a conclusive cytological result, whereas 31.8% (n=27) were non-diagnostic and required further evaluation with additional imaging, including computed tomography (CT) and/or magnetic resonance imaging (MRI), to establish a final diagnosis and guide clinical management (Figure 2).

Data are presented as percentage (%) and number (n). Statistical significance was considered at p<0.05.

Turnaround time

**Figure 3:**
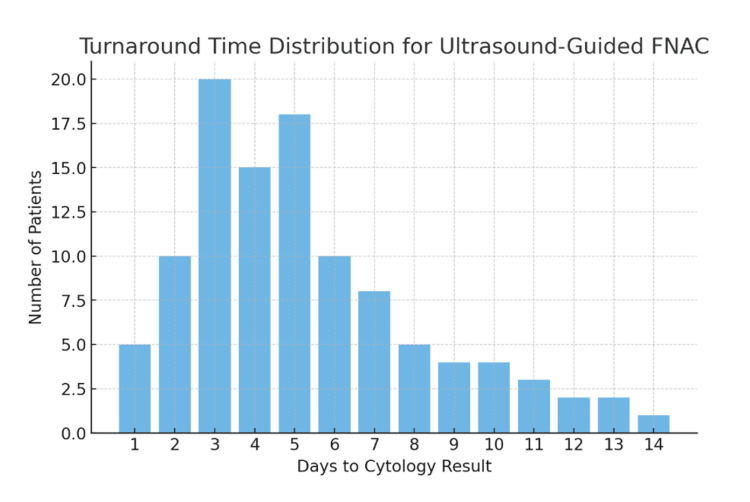
Turnaround time distribution for ultrasound-guided fine-needle aspiration cytology (FNAC). Data is expressed as number (N) of patients per day to cytology result (mean±SD). Statistical significance was considered at p<0.05.

The mean turnaround time from US-FNAC to receipt of the cytology report was 5.0±3.2 calendar days (range: 1-14 days), equivalent to a mean of 3.0±2.4 working days (range: 1-10 days) (Figure 3). This finding highlights the efficiency of the one-stop neck lump clinic model where clinical assessment, ultrasound examination, and tissue sampling are completed during a single patient visit.

A shorter diagnostic interval is critical in head and neck malignancy management, as it facilitates timely referral to the multidisciplinary team (MDT) and prompt initiation of definitive treatment.

Data are expressed as mean±standard deviation (SD). Statistical significance was considered at p<0.05.

Subsequent imaging

Post-US-FNAC imaging was performed in 89.9% (n=116) of patients. Of these, 34.1% (n=44) required further imaging due to insufficient initial cytology, and 20.9% (n=27) underwent additional imaging because, although the FNAC samples were cytologically adequate, the findings were non-diagnostic and did not yield a definitive diagnosis. In contrast, 10.1% (n=13) had diagnostic FNAC results sufficient to guide clinical management without further imaging.

The follow-up imaging modalities included ultrasound, CT, and MRI, with ultrasound being the most frequently utilised. The high rate of post-FNAC imaging reflects the comprehensive, multidisciplinary approach within the one-stop clinic model, ensuring that cases with either inadequate or inconclusive cytology underwent appropriate follow-up to confirm the diagnosis and guide treatment planning.

Data are presented as percentage (%) and number (n), with p<0.05 considered statistically significant.

## Discussion

Head and neck cancer represents a heterogeneous group of malignancies whose incidence has continued to rise over recent decades, with current UK estimates ranging from eight to 15 cases per 100,000 population [1]. Within England, marked regional variation in incidence has long been recognised, with persistently higher rates reported in northern regions [2], a trend that remains evident in recent data published by Public Health England [3].

Diagnostic delay and late initiation of treatment remain the major contributors to the comparatively lower survival outcomes observed among head and neck cancer patients in the UK relative to other European nations [4]. In response to these challenges, the National Institute for Health and Care Excellence (NICE) introduced guidance in 2004 aimed at improving the delivery of head and neck cancer services nationwide [5]. These recommendations advocated the establishment of one-stop neck lump clinics, modelled on the proven efficiency of one-stop breast clinics, and emphasised the use of fine-needle aspiration (FNA) for the evaluation of all suspicious neck masses to expedite diagnosis and streamline care [6].

Ultrasound is now widely recognised as the first-line imaging modality for assessing neck lumps, while obtaining a tissue diagnosis prior to treatment initiation remains integral to optimal patient management [7]. The diagnostic role of FNA in head and neck pathology dates back to the 1930s, first described by Martin and Ellis, and subsequently refined in later studies [8,9]. However, palpation-guided FNA fell out of favour owing to its high rate of inadequate samples, often resulting from suboptimal needle placement and inconsistent sampling of heterogeneous lesions [7-10]. The advent of ultrasound-guided FNA (US-FNA) has since addressed these limitations, providing real-time visualisation for precise needle positioning, minimising sampling error, and significantly improving diagnostic yield and accuracy [10-12]. Moreover, US-FNA enables the sampling of non-palpable or deep-seated lesions while avoiding necrotic or cystic areas that commonly produce non-diagnostic material [10-12].

FNA procedures may be undertaken by radiologists, cytopathologists, or trained surgeons. In a landmark series, Dongbin Ahn, Heejin Kim, Jin Ho Sohn, Jae Hyuk Choi, and Kyung Jin Na [13] evaluated the adequacy and diagnostic accuracy of surgeon-performed US-FNA across a spectrum of cervical pathologies. Among 617 procedures, the study demonstrated a sample adequacy rate exceeding 90%, with a diagnostic accuracy of 92%, sensitivity of 88%, and specificity of 98%. These results affirm that, with appropriate training and experience, surgeons can achieve diagnostic outcomes comparable to those of radiologists and cytopathologists.

Beyond diagnostic accuracy, surgeon-performed US-FNA offers several pragmatic advantages. Reported benefits include reduced patient waiting times, fewer appointments, improved continuity of care, and more rapid clinical decision-making within outpatient settings [13]. This approach may also be cost-effective, particularly in healthcare systems where radiology capacity is limited or where rapid diagnostic pathways are required [13].

The presence of an on-site cytopathologist during FNA remains the gold standard, as immediate sample assessment and preliminary diagnosis substantially enhance diagnostic reliability [7-12]. Real-time cytopathological evaluation allows direct correlation between clinical, imaging, and cytological findings, reducing diagnostic error and enabling immediate repeat sampling where necessary [7-12]. On-site cytology also facilitates ancillary investigations such as immunocytochemistry without additional patient visits [10,11]. However, this model is resource-intensive, with estimated UK staffing costs of approximately £81 per hour for a pathologist and £20 per hour for a cytology technician [10,11]. Furthermore, workforce shortages across many NHS pathology departments limit the availability of on-site cytology support [10,11]. Employing trained cytology technicians to prepare slides in clinic can improve sample adequacy, but does not provide immediate diagnostic feedback [10,11].

Under current NHS cancer waiting-time standards, patients must commence definitive treatment within 62 days of urgent general physician (GP) referral and within 31 days of confirmed diagnosis [4]. Given the two-week timeframe from referral to initial consultation, this leaves approximately 17 days for diagnosis and treatment planning [4]. The absence of on-site cytology can lengthen this interval; Horvath and Kraft [10] reported that up to 15% of patients required repeat biopsies, leading to significant delays in staging and initiation of therapy.

Integration of on-site cytology within surgeon-led one-stop clinics has been shown to improve diagnostic efficiency, reduce dependence on radiology departments, and support timely multidisciplinary decision-making. This model aligns with national cancer performance targets and represents a sustainable solution to workforce and service pressures within head and neck cancer diagnostics [3,7,10-12]. Where on-site cytology is unavailable, well-structured surgeon-led clinics remain capable of achieving high sample adequacy and efficient patient management [13].

This study has several strengths. It evaluates surgeon-performed US-FNAC in a real-world, one-stop neck lump clinic, reflecting a rapid and efficient clinical pathway that enhances continuity of care and expedites patient management. The study demonstrates that, with appropriate training and experience, surgeons can achieve high sample adequacy and diagnostic yield, supporting the feasibility of this model where on-site cytology may not be available.

However, several limitations should be acknowledged. The study’s retrospective, single-center design and relatively small sample size may limit the generalizability of the findings. The relatively low FNAC adequacy rate and the high rate of subsequent imaging also represent important constraints. In addition, detailed lesion-specific analyses, evaluation of operator variability, and comprehensive cytological grading were not performed. The absence of a direct comparison with radiologist- or cytopathologist-performed FNAC limits the ability to assess relative diagnostic accuracy and pathway efficiency. Future multicenter studies incorporating clearer cytology criteria, lesion-specific analysis, and a prospective or comparative design would strengthen the evidence base and allow the evaluation of cost-effectiveness, patient satisfaction, and long-term clinical outcomes.

These limitations are balanced by the study’s strengths in providing a practical, real-world evaluation of surgeon-performed US-FNAC within a rapid diagnostic pathway, offering important insights for service delivery and workforce planning.

## Conclusions

This study contributes to the growing evidence that head and neck surgeons, with appropriate training, can perform ultrasound-guided FNAC safely and with acceptable diagnostic adequacy in a real-world one-stop clinic setting. While on-site cytology remains the diagnostic gold standard, integrating surgeon-performed US-FNAC into streamlined clinics may help improve workflow efficiency and reduce dependence on radiology services, and support compliance with NHS cancer pathway targets. However, given the retrospective design, limited cytological detail, and absence of a comparator group, the conclusions regarding diagnostic performance and pathway efficiency should be interpreted with caution. Broader implementation may also be constrained by staffing and resource availability. Future multicenter prospective studies - including direct comparisons between surgeon-led and radiology-led FNAC pathways - are needed to more robustly evaluate diagnostic accuracy, cost-effectiveness, patient-centred outcomes, and their implications for service planning and policy development.

## References

[1] Gormley M, Creaney G, Schache A, Ingarfield K, Conway DI (2022). Reviewing the epidemiology of head and neck cancer: definitions, trends and risk factors. Br Dent J.

[2] McCarthy CE, Field JK, Rajlawat BP, Field AE, Marcus MW (2015). Trends and regional variation in the incidence of head and neck cancers in England: 2002 to 2011. Int J Oncol.

[3] Atlas of health variation in head and neck cancer in England. Gov.UK. London: Government Statistical Service.

[4] Neal RD (2009). Do diagnostic delays in cancer matter?. Br J Cancer.

[5] (2004). National Institute for Health and Clinical Excellence. Improving outcomes in head and neck cancers: Cancer service guideline. London: NICE. https://www.nice.org.uk/guidance/csg6.

[6] Schache A, Kerawala C, Ahmed O (2021). British Association of Head and Neck Oncologists (BAHNO) standards 2020. J Oral Pathol Med.

[7] Schwarz R, Chan NH, MacFarlane JK (1990). Fine needle aspiration cytology in the evaluation of head and neck masses. Am J Surg.

[8] Martin HE, Ellis EB (1930). Biopsy by needle puncture and aspiration. Ann Surg.

[9] El Hag IA, Chiedozi LC, al Reyees FA, Kollur SM (2003). Fine needle aspiration cytology of head and neck masses. Seven years' experience in a secondary care hospital. Acta Cytol.

[10] Horvath L, Kraft M (2019). Evaluation of ultrasound and fine-needle aspiration in the assessment of head and neck lesions. Eur Arch Otorhinolaryngol.

[11] Wagner JM, Monfore N, McCullough AJ, Zhao L, Conrad RD, Krempl GA, Alleman AM (2019). Ultrasound-guided fine-needle aspiration with optional core needle biopsy of head and neck lymph nodes and masses: comparison of diagnostic performance in treated squamous cell cancer versus all other lesions. J Ultrasound Med.

[12] Novoa E, Gürtler N, Arnoux A, Kraft M (2012). Role of ultrasound-guided core-needle biopsy in the assessment of head and neck lesions: a meta-analysis and systematic review of the literature. Head Neck.

[13] Ahn D, Kim H, Sohn JH, Choi JH, Na KJ (2015). Surgeon-performed ultrasound-guided fine-needle aspiration cytology of head and neck mass lesions: sampling adequacy and diagnostic accuracy. Ann Surg Oncol.

